# Linkage Evidence for a Two-Locus Inheritance of LQT-Associated Seizures in a Multigenerational LQT Family With a Novel *KCNQ1* Loss-of-Function Mutation

**DOI:** 10.3389/fneur.2019.00648

**Published:** 2019-06-25

**Authors:** Harald Prüss, Guido Gessner, Stefan H. Heinemann, Franz Rüschendorf, Ann-Kathrin Ruppert, Herbert Schulz, Thomas Sander, Wilhelm Rimpau

**Affiliations:** ^1^Department of Neurology and Experimental Neurology, Charité - Universitätsmedizin Berlin, Berlin, Germany; ^2^German Center for Neurodegenerative Diseases (DZNE) Berlin, Bonn, Germany; ^3^Department of Biophysics, Center for Molecular Biomedicine, Friedrich Schiller University Jena & Jena University Hospital, Jena, Germany; ^4^Max Delbrück Centre for Molecular Medicine, Berlin, Germany; ^5^Cologne Center for Genomics, University of Cologne, Cologne, Germany

**Keywords:** LQT syndrome, KCNQ1, familial epilepsy, risk haplotype, genetic modifier

## Abstract

Mutations in several genes encoding ion channels can cause the long-QT (LQT) syndrome with cardiac arrhythmias, syncope and sudden death. Recently, mutations in some of these genes were also identified to cause epileptic seizures in these patients, and the sudden unexplained death in epilepsy (SUDEP) was considered to be the pathologic overlap between the two clinical conditions. For LQT-associated *KCNQ1* mutations, only few investigations reported the coincidence of cardiac dysfunction and epileptic seizures. Clinical, electrophysiological and genetic characterization of a large pedigree (*n* = 241 family members) with LQT syndrome caused by a 12-base-pair duplication in exon 8 of the *KCNQ1* gene duplicating four amino acids in the carboxyterminal KCNQ1 domain (*KCNQ1*dup12; p.R360_Q361dupQKQR, NM_000218.2, hg19). Electrophysiological recordings revealed no substantial KCNQ1-like currents. The mutation did not exhibit a dominant negative effect on wild-type KCNQ1 channel function. Most likely, the mutant protein was not functionally expressed and thus not incorporated into a heteromeric channel tetramer. Many LQT family members suffered from syncopes or developed sudden death, often after physical activity. Of 26 family members with LQT, seizures were present in 14 (LQTplus seizure trait). Molecular genetic analyses confirmed a causative role of the novel *KCNQ1*dup12 mutation for the LQT trait and revealed a strong link also with the LQTplus seizure trait. Genome-wide parametric multipoint linkage analyses identified a second strong genetic modifier locus for the LQTplus seizure trait in the chromosomal region 10p14. The linkage results suggest a two-locus inheritance model for the LQTplus seizure trait in which both the *KCNQ1*dup12 mutation and the 10p14 risk haplotype are necessary for the occurrence of LQT-associated seizures. The data strongly support emerging concepts that *KCNQ1* mutations may increase the risk of epilepsy, but additional genetic modifiers are necessary for the clinical manifestation of epileptic seizures.

## Introduction

Prolonged QT intervals are the diagnostic hallmark of inherited long-QT (LQT) syndromes which comprise a growing number of mutations in ion channels, with the gene encoding the voltage-gated potassium channel KCNQ1 (Kv7.1) being the most common LQT gene (OMIM **#**192500: LQT1) (1). LQT syndrome by *KCNQ1* mutations has long been considered as a cardiac disease with the risk of ventricular arrhythmias and sudden cardiac death, resulting from prolongation of the cardiac action potential. However, patients with LQT syndrome can also present with epileptic seizures which suggested that the underlying genetic cause might be relevant for both phenotypes (2). In patients with LQT1 syndrome due to *KCNQ1* mutations, clinical seizures were consistently observed (3, 4). Indeed, recent research identified the dual expression of KCNQ1 in the heart and in the brain with neurons in forebrain networks and brainstem nuclei expressing the highest levels (5). The same study used a mouse model carrying a human *KCNQ1* knock-in mutation which resulted not only in cardiac arrhythmias but also in seizures and sudden unexpected death in epilepsy (SUDEP). The model reflects emerging evidence from clinical cohorts of patients with LQT syndrome in which there is an increased association with epilepsy and seizure-like phenotypes, in particular in the related LQT disease with mutations in the gene encoding the voltage-gated potassium channel KCNH2 (Kv11.1, hERG1) (6, 7).

More than 10 years ago, we have identified a family of Kurdish descent with a novel mutation in the *KCNQ1* gene, consisting of a 12-base-pair duplication (*KCNQ1*dup12) in exon 8 (8). However, it was unclear whether this mutation is causing functional effects, thus plausibly explaining the clinical symptoms. Remarkably, identification of the *KCNQ1* mutation in the index patients was antedated by a several years' history of epileptic seizures and treatment with anti-epileptic drugs including carbamazepine. Although suggestive, at that time it was an unsolved question whether *KCNQ1* mutations may cause both, cardiac symptoms and epileptic seizures. It was rather contrarily believed that epileptic seizures are only a consequence of cardiac arrhythmias, e.g., torsades de pointes (TdP), a polymorphic ventricular tachycardia potentially leading to cerebral hypoperfusion and seizures (9). For *KCNQ1* mutation carriers, only one small family having both, LQT syndrome and epilepsy, has been characterized in detail (10). Thus, further investigations are required to explore pleiotropic effects of *KCNQ1* mutations on cardiac symptoms together with epileptic seizures in patients with LQT1 syndrome. Therefore, we markedly extended the characterization of the Kurdish pedigree—one of the largest of a family with LQT syndrome—aiming at the correlation between the *KCNQ1*dup12 mutation and the clinical manifestation of variable symptoms, in particular syncopes, seizures, and sudden death (11).

## Methods

### Study Pedigree and Clinical Phenotype

After identification of the index case (IV57) (8), the family was systematically expanded (Supplementary Figure 1), resulting in 241 family members across five generations, for 233 of whom a detailed medical history was available. The pedigree consisted of 117 (48.5%) females and 124 (51.5%) males. The study was approved by the Charité University Hospital Institutional Review Board. All participants gave their written informed consent in accordance with the Declaration of Helsinki.

Determination of the clinical phenotype based on interviews with patients or close relatives, and was performed blinded to the genetic status of the subjects. Characteristic videos demonstrating the differences between syncope and seizures were used to train relatives in better explanation of witnessed events, particularly focusing on duration, aura symptoms, presence of automatisms, focal neurologic signs, and postictal confusion. EEG recordings (which are not the current standard of care for the evaluation of LQT syndrome) were not available for this Kurdish family.

### ECG Analysis

Available 12-lead ECG recordings were blindly analyzed by an experienced cardiologist. Measured QT intervals were corrected (QTc) for heart rates between 60 and 80 bpm according to Bazett's formula. For heart rates faster than 80 bpm, Fridericia's formula was used. As repeated ECGs were not available in most cases, LQTS was diagnosed in this study according to the Class IIa recommendation of the European Society of Cardiology (ESC) guidelines (12) by considering QTc times >450 ms in men and >460 ms in women pathologic.

### DNA Analyses

#### Sequence Analyses

DNA samples of 60 family members were extracted from whole blood. Initially, mutation screening of the affected members (IV57, V84) of the index family was performed by Sanger sequencing for an LQT candidate gene panel. Subsequently, whole-exome sequencing was applied for the index patient IV57. Exonic targets were enriched using the SeqCap EZ Human Exome Library v2.0 (Roche, NimbleGen). Sequencing was performed on an Illumina HiSeq2000, using a 100 bp paired-end read protocol.

#### Genotyping of the *KCNQ1* Mutation

Large-scale genotyping of the 12-bp duplication in exon 8 of the *KCNQ1* gene (*KCNQ1*dup12 variation: c.1068_1079delGCAGAAGCAGAGins(GCAGAAGCAGAG)2, NM_000218.2, p.R360_Q361insQKQR, ENSP00000155840, hg19) was carried out by fragment length assay (*KCNQ1*dup12 variation: genomic reference allele: 128 bp, mutation: 140 bp). Polymerase chain amplification (PCR) of the *KCNQ1*dup12 variation was performed using flanking fluorescence-labeled forward primer GAGCCTCCTGTCCATTCCTT and the reverse primer ACCGCACCTGAATGAGTGA. Fragment sizes of the biallelic PCR products were separated on ABI 3730 DNA Analyzers and genotypes scored using GENEMAPPER version 3.7 software (Applied Biosystems, Foster City, CA, USA).

#### Linkage Analyses

Genome-wide linkage mapping was carried out in this multigenerational family including 24 family members of whom 17 members were affected by LQT (**Figure 2A**) and 12 members also exhibited epileptic seizures (**Figure 2B**), using 5455 quality-filtered autosomal single nucleotide polymorphisms (SNPs) genotyped by the Illumina HumanLinkage-12 Genotyping BeadChip (Illumina Inc., San Diego, CA, USA). Parametric two-point and multipoint linkage analyses were performed for an affecteds-only classification assuming an autosomal dominant inheritance with a phenocopy rate of 1% and a mutation prevalence of 0.1%. The linkage program MLINK (13) was applied for parametric two-point and MERLIN for parametric multipoint linkage analysis (14). Genotype data quality control and data management was facilitated by the program ALOHOMORA (15). HaploPainter (16) was used for drawing pedigrees with haplotypes based on MERLIN calculations.

### Electrophysiology

For this study, expression constructs of *KCNQ1* or *KCNH2* in pCDNA3 (Invitrogen) and *KCNE1* in pEGFP-N1 were used. Mutation *KCNQ1*dup12 and *KCNH2*-R878C were introduced by overlap-extension PCR and verified by sequencing. HEK293T cells were maintained with 5% CO_2_ at 37°C in 45% DMEM + 45% F12 + 10% FCS. Cells were plated on glass coverslips and transfected 24 h later with a constant amount of plasmid DNA encoding wild-type or mutant KCNQ1, or a 1:1 mixture. CD8-encoding DNA (<20% of channel DNA) was cotransfected, allowing identification of transfected cells via CD8-detecting beads (Dynabeads, Life Technologies). For coexpression of *KCNE1*, an equal amount of DNA encoding C-terminally GFP-tagged *KCNE1* was added. In these cases, transfected cells were identified by fluorescence microscopy. Whole-cell patch-clamp measurements were performed with an EPC9 amplifier, operated with PatchMaster software (HEKA Elektronik, Lambrecht, Germany), 2 days after transfection. The bath solution contained (in mM): 146 NaCl, 4 KCl, 2 MgCl_2_, 2 CaCl_2_, 10 HEPES (pH 7.4, NaOH). The pipette solution consisted of (in mM): 130 KCl, 2 Mg-ATP, 10 EGTA, 10 HEPES (pH 7.4, KOH). Data were analyzed with FitMaster (HEKA Elektronik) and IgorPro (Wavemetrics, Lake Oswego, OR, USA).

## Results

### Family Members

**Figure 1:**
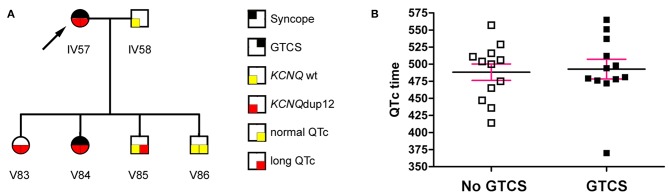
**(A)** The index patient (arrow) had a long-standing history of seizures. Diagnosis of LQT syndrome was established when her daughter V84 was diagnosed with a prolonged QT interval after two syncopes. **(B)** The QTc interval (mean ± SEM) was not significantly different between mutation carriers with or without generalized tonic-clonic seizures (GTCS) (*P* = 0.795, Mann Whitney test).

The initial diagnosis of LQT syndrome was established in the index patient IV57 (Figure 1A, arrow) when her daughter V84 was diagnosed with a prolonged QT interval after two syncopes. Before that, the index patient had been diagnosed of epilepsy already for more than 20 years. In retrospect, however, several of the “spells” in this patient during adolescence were due to cardiac affection including palpitations, exercise-induced syncope, presyncopal aura, short-lasting unconsciousness with rapid reorientation, most frequently after awakening in the morning. In adulthood, the patient suffered mainly from generalized tonic-clonic seizures (GTCS), prolonged postictal disorientation, enuresis and tongue bite, resulting in treatment with carbamazepine. Treatment with β blockers resulted in complete remission of cardiac symptoms and partial remission of epilepsy (8).

Following this initial observation, we next analyzed the variable clinical symptoms in one of the largest pedigrees reported of a family with LQT syndrome (Supplementary Figure 1). The expanded pedigree contained 241 family members across five generations. LQT syndrome appeared in the family branches of the founder III24 (Supplementary Figure 1, arrow) with his three marriages (III23, III25, and III27), following an autosomal-dominant segregation pattern. Family history of sudden death was common, 19 children died of SIDS (sudden infant death syndrome) or SUDEP. For example, in one family tree (IV53/IV54) 4 of 8 children died from sudden death at 0–1 years of age.

Aiming primarily at the clinical distinction between symptoms of cardiac syncopes vs. epileptic seizures and due to the lack of available EEGs in this Kurdish pedigree, we interviewed patients and relatives in a standardized way blinded to the genetic status, supported by teaching videos of representative syncope or seizure events. We identified 24 family members with symptoms compatible with syncope, 6 of which passed away at 0–12 years of age. Of the remaining 18, 16 (88.9%) showed prolonged QTc intervals. Clinical assessment revealed 24 family members with a history of GTCS including eight deaths at 0–12 years of age. Two family members had GTCS with normal (V51) or undetermined (V52) QTc intervals, while all remaining 14 patients had GTCS in the context of ECG-confirmed prolonged QTc time (LQTplus seizure trait) (Supplementary Figure 1). However, the length of the QTc interval in mutation carriers was not different between family members with or without GTCS (Figure 1B) (*P* = 0.795, Mann Whitney test). Also, GTCS frequency and QTc time did not show a significant correlation (*P* = 0.18, *R*^2^ = 0.32, *n* = 7 family members), collectively suggesting that seizures were not a result of more severe cardiac dysfunction. All 14 patients experienced both types of paroxysmal events, namely those related to cardiac dysfunction (such as with profound pallor at time of spells or exercise-related events) as well as spontaneous epileptic seizures without antecedent cardiac symptoms (e.g., starting during rest and followed by prolonged postictal confusion).

### Mutation Screening

Initial Sanger sequencing of the most common LQT genes in the index patient IV57 identified a novel heterozygous 12-bp-duplication in exon 8 of the *KCNQ1* gene (*KCNQ1*dup12; chr11:2606477-2606488(GCAGAAGCAGAG>(GCAGAAGCAGAG)_2_, hg19) duplicating four amino acids in the carboxyterminal KCNQ1 domain (p.R360_Q361dupQKQR, NM_000218.2, hg19) (8). The *KCNQ1*dup12 mutation was not detected in gnomAD in 123,136 exomes and 15,496 genomes from unrelated individuals (http://gnomad.broadinstitute.org/). Complementary whole-exome sequencing was carried out in the index patient IV57 affected by the LQTplus seizure trait to search for additional mutations in a panel of 13 genes causing cardiac arrhythmia. We were able to confirm the *KCNQ1*dup12 mutation and detected a rare heterozygous missense variant in exon 9 of *KCNH2*, the second most common LQT gene (LQT2: OMIM #613688; dbSNP: rs370393086; chr7:150647022G>A, c.2632C>T, NM_172056.2, p.R878C, CCDS47747.1, hg19). However, protein function prediction did not indicate a substantial functional alteration of the amino acid substitution (p.R878C, ENST00000430723, Polyphen: benign). Electrophysiological characterization of the mutant channels hERG1-R878C upon heterologous expression in HEK293T cells did not reveal functional differences compared to wild-type hERG1 (Supplementary Figure 3). Moreover, this rare *KCNH2* missense variant (p.878C) originated from the second wife (III25) of the *KCNQ1*dup12 mutation founder (III24) and therefore its segregation was restricted to this family branch. Although the rare *KCNH2* missense variant was present in the putative LQT phenocopy lacking the *KCNQ1*dup12 mutation (V85), we did not observe a familial cosegregation with the LQT- or LQTplus seizure trait. Thus, this rare *KCNH2* missense variant or other common LQT genes are unlikely to exert a substantial pathogenic effect on the LQT trait in this family.

### Strong Linkage Between the *KCNQ1*dup12 Mutation and Both the LQT Trait and LQTplus Seizure Trait

**Figure 2:**
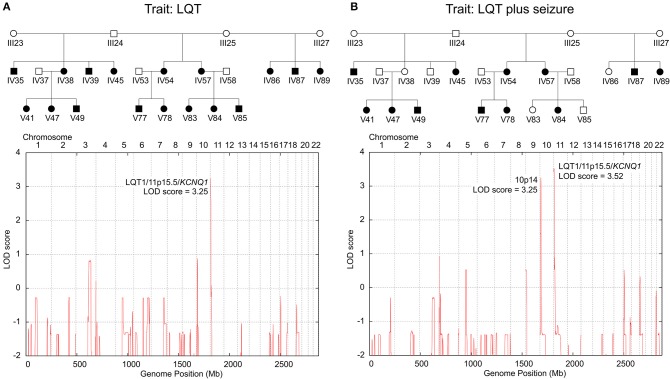
Genome-wide parametric multipoint linkage analyses for two LQT-trait models. **(A)** Linkage results for the LQT trait only identified a single major LQT-locus at 11p15.5/*KCNQ1*. **(B)** Linkage results for the LQT-associated seizure trait revealed cosegregating susceptibility loci at 11p15.5/*KCNQ1* and 10p14, suggesting a dominant-dominant two-locus inheritance model. Blackened symbols in the pedigree denote individuals affected by the investigated LQT-trait model (**A**: LQT only; **B**: LQT plus epileptic seizure).

Genotyping of the *KCNQ1*dup12 variation in DNA samples of 60 family members detected the always heterozygous *KCNQ1*dup12 mutation in 24 out of 25 genotyped family members with LQT syndrome and all 14 genotyped family members affected by both LQT syndrome and epileptic seizures (LQTplus seizure trait). One family member (V85) with LQT syndrome did not carry the *KCNQ1*dup12 mutation and has to be considered as putative phenocopy. Overall, 24 out of 29 *KCNQ1*dup12 mutation carriers were affected by an LQT syndrome, suggesting an incomplete penetrance of about 80%. Two-point parametric linkage analysis including 68 family members showed significant evidence for linkage between the *KCNQ1*dup12 mutation and the LQT trait (25 affected individuals) assuming an autosomal dominant inheritance with a phenocopy rate of 1% based on the affected individuals only (maximum LOD score: 5.21 at a recombination fraction (θ) of 0.03, *P* = 4.83 × 10^−7^). Likewise, significant evidence for linkage was obtained between the *KCNQ1*dup12 mutation and the LQTplus seizure trait in 14 affected family members (maximum LOD score: 4.12 at *KCNQ1, P* = 6.63 × 10^−6^). To search for other potential LQT loci, we carried out genome-wide parametric multipoint linkage analysis in 24 family members including 17 members with LQT. For an affecteds-only classification and autosomal dominant mode-of-inheritance with a presumed phenocopy rate of 1%, we found a single significant linkage peak at the LQT1/*KCNQ1* locus (multipoint LOD score: 3.25, *P* = 5.47 × 10^−5^; Figure 2A).

### Linkage Evidence for an LQT1/*KCNQ1* Cosegregating 10p14 Risk Haplotype Suggesting a Two-locus Inheritance Model for the LQTplus Seizure Trait

To examine whether the LQTplus seizure trait may reflect a variable expressivity of the *KCNQ1*dup12 mutation or whether additional genetic modifier loci are involved, we carried out genome-wide parametric multipoint linkage analyses for an affecteds-only classification using 5455 autosomal SNPs. For the LQTplus seizure trait (12 affected family members), we detected two significant linkage peaks, one predominant peak at the LQT1/*KCNQ1* locus in the chromosomal region 11p15.5–p15.4 (multipoint LOD score: 3.52, *P* = 2.83 × 10^−5^, Supplementary Figure 4A) and the second linkage peak in the chromosomal region 10p14 (multipoint LOD score = 3.25 at rs1033912, chr10:9592521, *P* = 5.47 × 10^−5^, Supplementary Figure 4B) (Figure 2B). The linkage results support a two-locus inheritance model presuming that both the major LQT1/*KCNQ1* locus and the modifier locus on chromosome 10p14 are necessary for the coincidental phenotypic expression of LQT and epileptic seizures in the present family. Haplotype analysis of the chromosomal region 10p14 revealed a risk haplotype spanning 3.8 Mb flanked by the SNPs rs2671303 and rs1535976 (chr10:8074577-11850132, hg19) (Supplementary Figure 5). Notably, all 12 family members affected by the LQTplus seizure trait carried both the *KCNQ1*dup12 mutation and the 10p14 risk haplotype. In addition, two family members (IV38 and IV39) also carried both the *KCNQ1*dup12 mutation and the 10p14 risk haplotype but exhibited only a severely prolonged LQT interval (>500 ms) without a known history of cardiac syncopes or epileptic seizures. This finding suggests an incomplete penetrance of approximately 80% for the two-locus model.

### Electrophysiology

**Figure 3:**
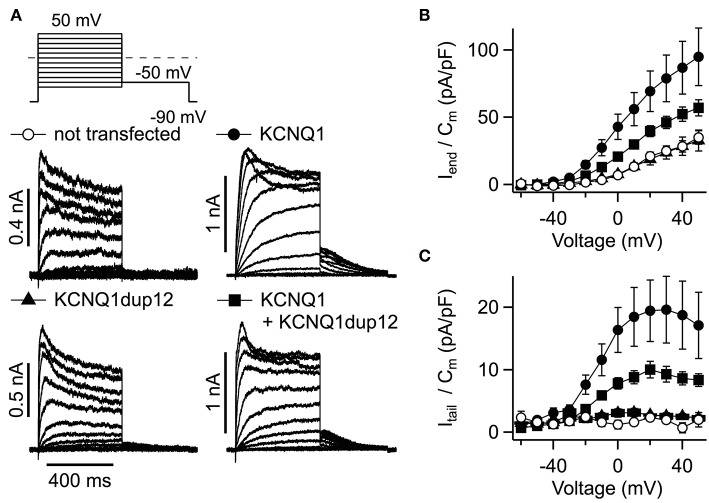
Functional impact of the *KCNQ1*dup12 mutation on KCNQ1-mediated K^+^ currents. **(A)** Superimposed whole-cell current recordings evoked by the pulse protocol shown on top. HEK293T cells were transfected with equal amounts of DNA encoding wild-type or mutant *KCNQ1* or a mixture of both, as indicated. Non-transfected cells served as controls. Current amplitude at the end of the depolarization (I_end_) and maximal outward tail current (I_tail_) amplitudes were normalized to the cell capacitance (C_m_), averaged and plotted against voltage in **(B,C)**, respectively. Data points are mean ± SEM (*n* = 8). Straight lines connect data points for clarity.

To assess the functional impact of the LQT mutation, the mutation was engineered into a *KCNQ1-*containing plasmid construct suited for expression in mammalian cells. As shown in Figure 3, *KCNQ1*dup12-expressing cells exhibited voltage-dependent, partially inactivating outward currents similar to currents observed from non-transfected control cells. Cells transfected with *KCNQ1* or an equimolar mixture of wild-type and mutant *KCNQ1* DNA exhibited stronger outward currents. Current amplitudes at the end of the depolarizations were determined, normalized to the cell capacitance, and plotted against voltage (Figure 3B; *n* = 8, each). For positive voltages, current densities of *KCNQ1*-expressing cells were significantly larger than for *KCNQ1*dup12-expressing cells or untransfected cells, whereas the latter two were indistinguishable. Upon coexpression of *KCNQ1*dup12, current densities were slightly but not statistically significantly smaller than that of cells expressing wild-type only. Since the outward currents at the end of the depolarizations are contaminated by endogenous channels, we also analyzed tail currents upon repolarization to −50 mV. *KCNQ1*-expressing cells exhibited substantial outward tail currents, characteristic for the slow gating of KCNQ1 channels. For *KCNQ1*dup12 or in untransfected cells, similar currents were barely detectable. Tail current densities from mutant plus wild-type transfected cells were slightly but not statistically significantly smaller than those from cells expressing wild-type only. This finding was reproduced in a second batch of cells with *n* = 15 cells each (Supplementary Figure 2), suggesting that mutation KCNQ1dup12 does not exhibit a dominant negative effect on KCNQ1 channels. To analyze the channel's voltage dependence, tail current vs. voltage data were fitted with a Boltzmann function. The resulting voltages for half-maximal activation (V_0.5_) were not significantly different between KCNQ1 (−14 ± 2 mV) and KCNQ1 plus mutant (−14 ± 1 mV, *P* = 0.76). The corresponding voltages for e-fold change in activation (V_e_) were also indistinguishable (9.0 ± 0.5 mV vs. 8.8 ± 0.6 mV, *P* = 0.81).

**Figure 4:**
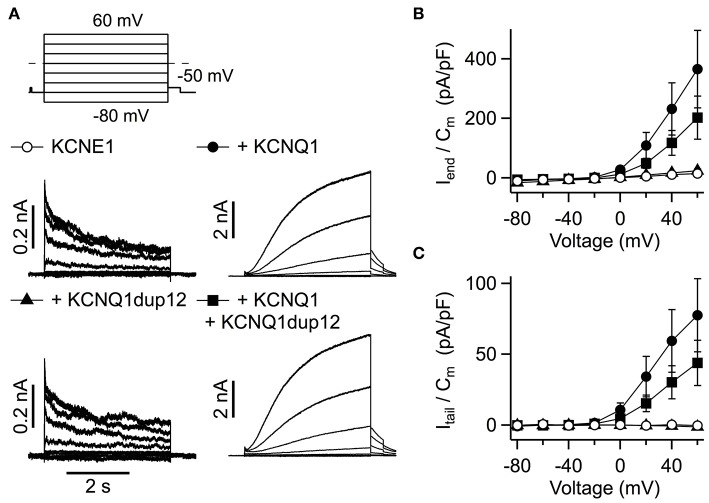
Functional impact of the *KCNQ1*dup12 mutation on I_Ks_ currents. **(A)** Superimposed whole-cell current recordings evoked by the pulse protocol shown on top. HEK293T cells were transfected with equal amounts of DNA encoding wild-type or mutant *KCNQ1* or a mixture of both, as indicated. An equal amount of GFP-tagged *KCNE1*-encoding DNA was cotransfected. Current amplitude at the end of the depolarization (I_end_) and maximal outward tail current (I_tail_) amplitudes were normalized to the cell capacitance (C_m_), averaged and plotted against voltage in **(B,C)**, respectively. Data points are mean ± SEM (*n* = 15). Straight lines connect data points for clarity.

We also assessed the functional impact of mutation KCNQ1dup12 in heteromeric KCNQ1 + KCNE1 channels, which resemble cardiac I_Ks_. Cells were transfected with equal amounts of *KCNQ1* DNA and *KCNE1* DNA. KCNE1 was N-terminally fused to GFP allowing identification of transfected cells. Cells transfected with *KCNQ1* and *KCNE1* exhibited I_Ks_-like currents, characterized by extremely slow activation upon depolarization (Figure 4A). Such types of outward currents were not observed in cells transfected with *KCNQ1*dup12 plus *KCNE1* or *KCNE1* alone. The latter exhibited fast activating and partially inactivating currents with indistinguishable current densities determined at the end of the depolarizations (Figure 4B). Upon repolarization to −50 mV, only marginal outward currents were observed. In contrast, *KCNQ1*-expressing cells exhibited robust outward tail currents. Maximal outward tail current densities (I_tail_/C_m_) are plotted against voltage in Figure 4C. Cotransfection of *KCNQ1*dup12 did not significantly diminish these current densities. To analyze the voltage dependence of activation, tail current vs. voltage data of those cells clearly differing from those expressing *KCNE1* only, were fitted with a Boltzmann function. Resulting V_0.5_ and V_e_ data were not significantly different between *KCNQ1* plus *KCNE1* expressing cells (V_0.5_: 30.0 ± 3.1 mV and V_e_: 13.7 ± 1.0 mV, *n* = 9) and those cells transfected with *KCNQ1*dup12 plus *KCNQ1* plus *KCNE1* (V_0.5_: 34.7 ± 3.4 mV (*P* = 0.33) and V_e_: 16.2 ± 0.9 mV (*P* = 0.88, *n* = 6).

## Discussion

While the clinical overlap between cardiac arrhythmias and epilepsy has been well-established for LQT-associated channelopathies related to the *SCN5A* and *KCNH2* genes (5, 7, 17), and epileptic seizures were found in 12% of patients with LQT syndrome associated with *KCNQ1* mutations (4), little is known about the detailed mechanisms and additional genetic risk factors leading to coincidental affection of heart and brain. Experimental murine data support the concept that *KCNQ1* mutations can directly lead to seizures, given the protein expression also in the brain (5). A recent study used next-generation sequencing in epilepsy patients with cardiac dysfunction or sudden death to explore the role of already known and novel candidate genes associated with epilepsy and SUDEP (18). Data revealed five new variants including one copy number variant in KCNQ1 (18).

Given that *KCNQ1* gene mutations account for up to 48% of congenital LQT syndromes (LQT1, OMIM: #192500) and that our functional analyses confirm a deleterious functional effect of the novel *KCNQ1*dup12 mutation, our sequence and linkage analyses provide striking evidence that the mutation plays a causative role for the LQT trait in this family. Epileptic seizures were common in the present cohort (LQTplus seizure trait) and frequently appeared independent of cardiac symptoms. Since about 5–10% of LQT patients carry mutations in more than one of the known LQT genes and typically express more severe phenotypes with a younger age at onset (19, 20), we addressed the question whether the LQTplus seizure trait may be explained by variable expressivity of the *KCNQ1*dup12 mutation or alternatively whether a genetic modifier locus besides *KCNQ1* contributes to the coincidence of LQT and seizures. Extensive search for additional mutations in known LQT genes detected a rare heterozygous missense variant of *KCNH2* in exon 9. *KCNH2* is the second most common LQT gene, and a loss-of-function mutation was associated with epilepsy and sudden death in a family with long QT syndrome (7). However, protein function was not significantly different from wild-type, and the segregation restricted to a small family branch makes a substantial pathogenic effect on the LQT trait in this family highly unlikely.

The manifestation of epileptic seizures also without precedent cardiac arrhythmias in all patients with LQTplus seizures and the absence of family members with epileptic seizures without LQT syndrome implicate the involvement of a genetic modifier. Indeed, genome-wide parametric multipoint linkage analyses revealed a second linkage peak in the chromosomal region 10p14, which was present in all 12 family members exhibiting the LQTplus seizure trait. In turn, *KCNQ1* mutation carriers did not have GTCS when lacking the cosegregating 10p14 haplotype. The findings suggest a dominant-dominant two-locus inheritance model in which the major LQT1/*KCNQ1* locus at 11p15.5–p15.4 and the modifier locus at 10p14 act together to express the LQTplus seizure trait (Figure 2B; Supplementary Figures 4, 5). The 10p14 risk haplotype encompasses 24 genes, of which the gene encoding the CUG triplet repeat, RNA-binding protein 2 (gene symbol: *CELF2*, chr10:10838851-11378674, Entrez 10659, hg19) represents a high-ranking candidate gene for the phenotypic expression of LQT-associated seizures. *CELF2* is part of CUG-BP, Elav-like family (*CELF1-6*) and is involved in the regulation of alternative RNA splicing in the brain (21). Mice deficient for *Celf4* exhibit a complex seizure disorder that includes both convulsive and non-convulsive seizures resulting from an increased neuronal excitation due to aberrant Na_v_1.6 (*Scn8a*) sodium channel activity (22). Likewise, haploinsufficiency of human *CELF4* has been implicated to cause an impaired neurodevelopment with febrile seizures in a patient with a truncated *CELF4* gene due to a *de novo* translocation (23). Together, these findings support an involvement of CELF proteins in the regulation of neuronal excitability and epileptogenesis. Notably, whole-exome sequencing in the index patient IV57 did not identify any deleterious coding mutation of the *CELF2* gene or other genes located on the risk haplotype on chromosome 10p14. However, mutations in the non-coding sequences of the *CELF2* gene may have been missed by targeted sequencing of the *CELF2* exons and exon-intron boundaries only.

Recurrent events in the patients of our cohort resulted from two different etiologies. First, they had LQT-related cardiac dysfunctions such as ventricular tachyarrhythmia or syncope. Secondary brain symptoms may be misdiagnosed as epileptic seizures but in fact result from hypoxia-triggered seizures. According to the diagnostic manual of the ILAE's (International League Against Epilepsy) commission on classification and terminology, these paroxysmal events are classified as epilepsy imitators. We reduced the common difficulties in clinical distinction between epileptic seizures and cardiac syncopes by use of representative videos and a detailed history paying particular attention to symptoms which help to distinguish both conditions (such as profound pallor at time of seizures or exercise-related events pointing to cardiac disease).

Second, the family members with LQT-associated seizures exhibited a genetic epilepsy related to the major LQT1/*KCNQ1* locus and the cosegregating 10p14 modifier locus, independent of cardiac dysfunction. Thus, the *KCNQ1*dup12 mutation represented a susceptibility gene not leading to GTCS alone, but caused seizures in combination with the 10p14 risk allele in this LQT1 family. This notion is further supported by clinical observations, including witnessed prolonged grand mal status of 20–30 min in one woman or witnessed GTCS in another by an epileptologist leading to treatment and assessment for epilepsy surgery. It is also supported by equal QTc intervals in mutation carriers with and without GTCS, suggesting that more severe heart disease is not the trigger for seizures.

Electrophysiological recordings with the here described *KCNQ1*dup12 mutation exhibited no channel activity of the mutant. In the absence or presence of KCNE1 subunits, we did not find substantial KCNQ1- or I_Ks_-like currents types significantly differing in current densities between *KCNQ1*dup12-transfected cells and their respective controls. This may be explained either by a lack of functional mutant protein, e.g., due to an impaired trafficking, or a complete loss of function, induced by the mutation. In the absence of further evidence for presence of the mutant protein in the plasma membrane, we cannot distinguish between these possibilities. Nevertheless, our data clearly indicate that the mutation does not exhibit a dominant negative effect. Neither with nor without coexpression of *KCNE1*, expression of the mutant significantly suppressed functional wild-type KCNQ1. Since in both cases coexpression of *KCNQ1*dup12 did not affect the voltage dependence of the channels, it appears more likely that the mutation impairs functional expression and thus the protein is not incorporated into a heteromeric channel tetramer.

Taken together, we electrophysiologically and genetically characterized a novel *KCNQ1* loss-of-function mutation in the largest family with LQT1 syndrome reported so far, demonstrating the causative role of the *KCNQ1*dup12 mutation for the LQT trait. In addition, we established a genetic two-locus model explaining the coincidental manifestation of LQT-associated epileptic seizures, based on a cosegregation of the *KCNQ1*dup12 mutation with a strong genetic modifier located in the chromosomal region 10p14. Our present findings provide novel insights into how *KCNQ1* mutations in LQT patients can lead to both, cardiac dysfunction with arrhythmias or syncope and increased cerebral excitability with epileptic seizures. They emphasize complex genetic models underlying genetic epilepsies beyond rare single gene effects to explain the remarkably variable phenotypic expressivity across familial epilepsies.

## Ethics Statement

The study was approved by the Charité University Hospital Institutional Review Board. All participants gave their written informed consent in accordance with the Declaration of Helsinki.

## Author's Note

The authors wish to honor in memory the late Prof. Dieter Janz. His careful attention of patient histories and pioneering genetic studies in epilepsy shaped the present work in all ranks.

## Author Contributions

HP, TS, and WR: design and conceptualization of the study. TS, HS, and FR: linkage and haplotype analyses. TS, HS, and A-KR: exome sequencing and variant genotyping. All authors: analysis or interpretation of the data, drafting, and revising the manuscript for intellectual content.

### Conflict of Interest Statement

The authors declare that the research was conducted in the absence of any commercial or financial relationships that could be construed as a potential conflict of interest.
