# Supplementary material for: Linkage Evidence for a Two-Locus Inheritance of LQT-Associated Seizures in a Multigenerational LQT Family With a Novel KCNQ1 Loss-of-Function Mutation
Source: Front Neurol. 2019 Jun 25;10:648. doi: 10.3389/fneur.2019.00648 (PMC6603176; doi:10.3389/fneur.2019.00648)

**Supplementary Material**

**Original Research Article**

**Linkage evidence for a two-locus inheritance of LQT-associated seizures in a multigenerational LQT family with a novel *KCNQ1* loss-of-function mutation**

Harald Prüss^1,2^, Guido Gessner^3^, Stefan H. Heinemann^3^, Franz Rüschendorf^4^, Ann-Kathrin Ruppert^5^, Herbert Schulz^5^, Thomas Sander^5^, Wilhelm Rimpau^6,^*

*Correspondence to:

Harald Prüss, MD

German Center for Neurodegenerative Diseases (DZNE) Berlin

c/o Charité – Universitätsmedizin Berlin

CharitéCrossOver (CCO), R 4-334, Charitéplatz 1, 10117 Berlin, Germany

Phone: +49 (0) 30 / 450-560399, Fax: +49 (0) 30 / 450-539916, [harald.pruess@charite.de](mailto:harald.pruess@charite.de)

Suppl. Figure 1. Clinical evaluation of the multigenerational LQT family including 241 members.

*See attached PDF*

Suppl. Figure 2.

(A) Superimposed whole-cell current recordings evoked by the indicated pulse protocol. HEK293T cells were transfected with equal amounts of DNA encoding KCNQ1 (red) or the mutant KCNQ1dup12 (black) or a mixture of both (green). Current amplitude at the end of the depolarization (I_end_) and maximal outward tail current (I_tail_) amplitudes were normalized to the cell capacitance (C_m_), averaged and plotted against voltage in (B) and (C), respectively. Data points are mean ± SEM (*n* indicated in panel B). Straight lines connect data points for clarity.


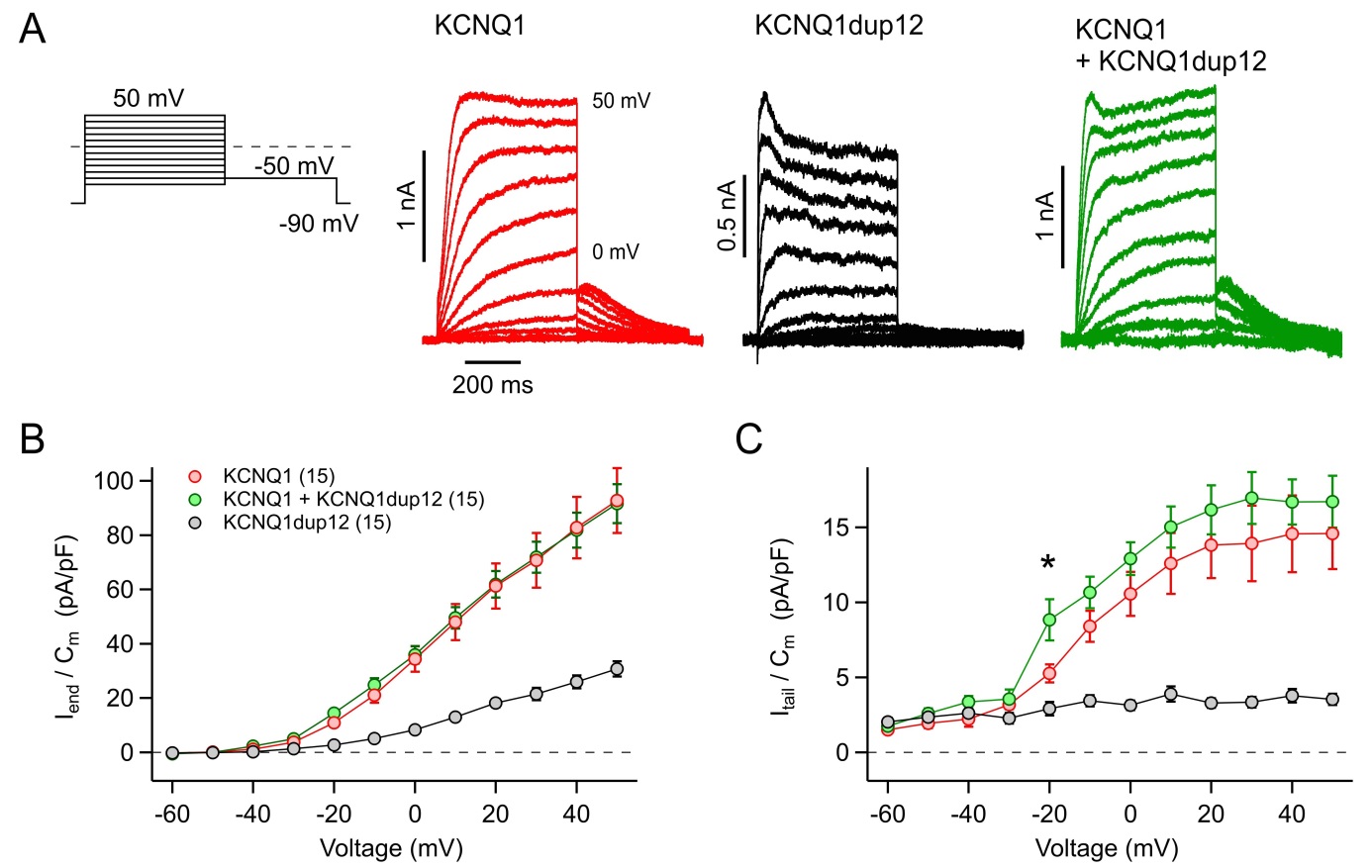


Suppl. Figure 3. Functional impact of the *R878C* mutation on hERG1 currents


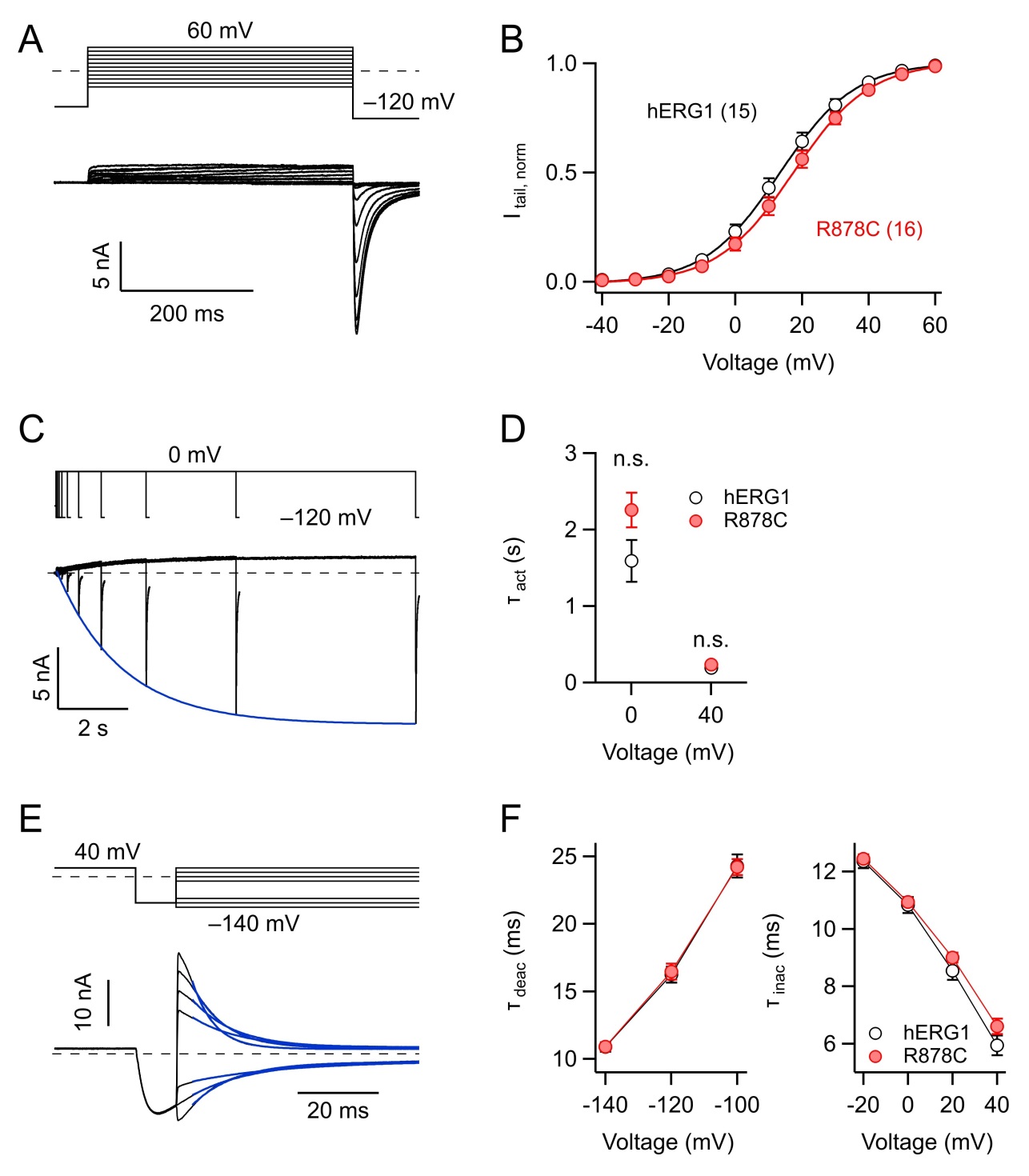
HEK293T cells were transfected with equal amounts of DNA encoding wild-type (wt) or mutant hERG1 (R878C). (A) Superimposed whole-cell current recordings evoked by the indicated pulse protocol. (B) Maximal inward tail-current amplitudes upon hyperpolarization to –120 mV (I_tail_) were plotted against voltage and fitted with a Boltzmann function. Resulting voltages for half-maximal activation (V_0.5_) were 13.4 ± 1.9 mV (wt) and 17.2 ± 1.9 mV (R878C) and voltages resulting in an e-fold conductance change (V_e_) were 10.2 ± 0.2 mV (wt) and 10.3 ± 0.1 mV. (C) Superposition of current traces recorded to analyze the activation time course. Maximum inward tail-current amplitudes upon hyperpolarization to –120 mV following depolarizing steps of increasing length were plotted against depolarization length and fitted monoexponentially (superimposed in blue). Averaged resulting time constants were not significantly different between wild type and R878C (D). (E) Superposition of current traces to analyze deactivation and inactivation kinetics: channels were fully opened by a short hyperpolarizing pulse after a prolonged depolarization. Channel inactivation and deactivation was then quantified by monoexponential fits (superimposed in blue) to the current decay at very positive and very negative voltages, thus favoring channel inactivation and deactivation, respectively. (F) Mean time constants of channel deactivation and inactivation (right) as a function of voltage. Straight lines connect data points for clarity. All current traces shown are from KCNH2-R878C expressed in HEK293T cells. Data points are mean ± SEM (*n* indicated in panel B).

Suppl. Figure 4.

Regional parametric multipoint linkage results for the LQTplus seizure trait revealed (A) a major locus at 11p15.5-p15.4 encompassing the LQT-associated *KCNQ1*dup12 mutation and (B) a potential modifier locus at 10p14, assuming an autosomal dominant inheritance model. Accordingly, our linkage results suggest a two-locus inheritance model for LQT-associated seizures. The linkage candidate region at 10p14 harbors the gene encoding the CUG triplet repeat, RNA-binding protein 2 (gene symbol: *CELF2*) which represents a high-ranking candidate gene for the LQT-associated seizure trait (B).


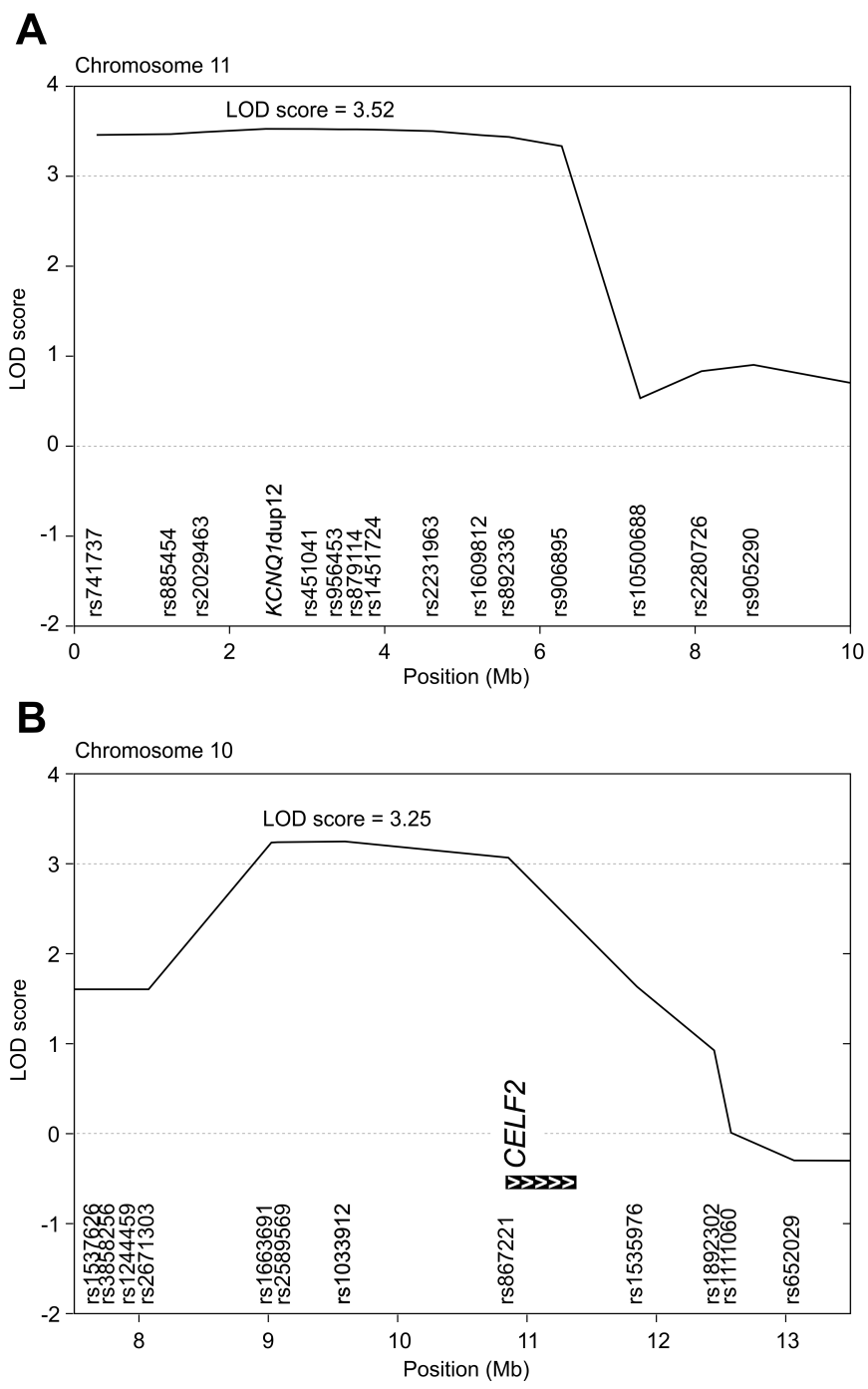


Suppl. Figure 5.

Two-locus inheritance model of the LQT-associated seizure trait composed by the cosegregation of a major risk haplotype at 11p15.5-p15.4 harboring a novel LQT-associated *KCNQ1*dup12 loss-of-function mutation and a potential modifier risk haplotype at 10p14 encompassing the candidate gene *CELF2*. The *KCNQ1*dup12 mutation is designated as allele 2 in the 11p15.5 risk haplotype. The 10p14 risk haplotype cosegregating with the LQT-associated seizure trait is highlighted by the black vertical bar.


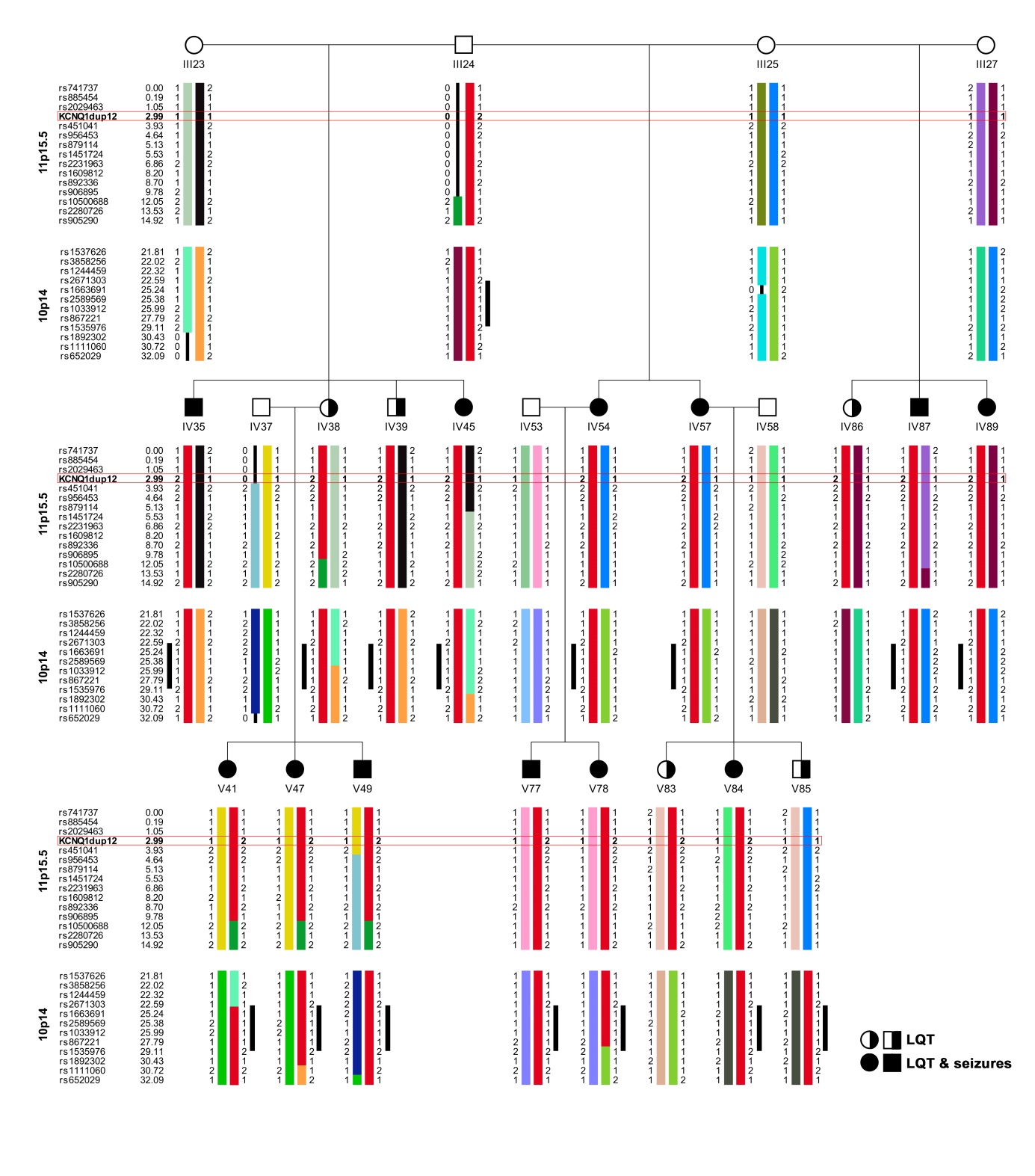

Supplement: Supplementary file 1 [file Data_Sheet_1.docx]
