# Supplementary figures and images for: Linkage Evidence for a Two-Locus Inheritance of LQT-Associated Seizures in a Multigenerational LQT Family With a Novel KCNQ1 Loss-of-Function Mutation
Source: Front Neurol. 2019 Jun 25;10:648. doi: 10.3389/fneur.2019.00648 (PMC6603176; doi:10.3389/fneur.2019.00648)

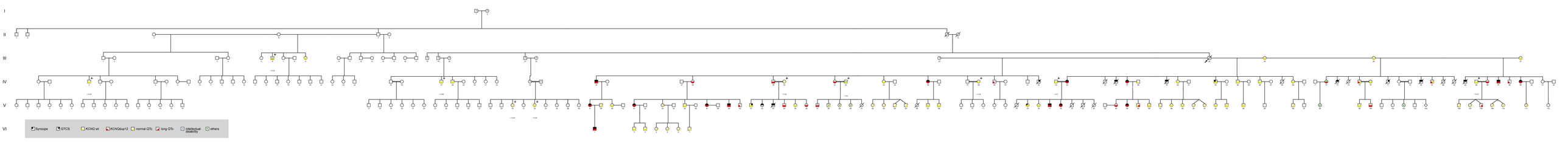

Supplement: Supplementary file 2 [file Image_1.pdf]
